# The impact of restoration methods for *Solidago*-invaded land on soil invertebrates

**DOI:** 10.1038/s41598-022-20812-5

**Published:** 2022-10-05

**Authors:** Peliyagodage Chathura Dineth Perera, Iwona Gruss, Jacek Twardowski, Cezary Chmielowiec, Magdalena Szymura, Tomasz H. Szymura

**Affiliations:** 1 Institute of Agroecology and Plant Production, Wrocław University of Environmental and Life Sciences, Grunwaldzki Sq 24a, 50-363 Wrocław, Poland; 2 Department of Plant Protection, Wrocław University of Environmental and Life Sciences, Grunwaldzki Sq 24a, 50-363 Wrocław, Poland; 3 Department of Ecology, Biogeochemistry and Environment Protection, University of Wrocław, Stanisława Przybyszewskiego 63, 51-148 Wrocław, Poland

**Keywords:** Environmental impact, Agroecology, Biodiversity, Invasive species

## Abstract

The belowground community structure of soil biota depends on plant composition and may be affected by invasive plant species. We hypothesized that the type of land restoration method applied affects the abundance and composition of soil invertebrates. Our field experiment centred on *Solidago* species control using different seed mixtures and methods of seed introduction (sowing mixtures: grasses, grasses with legumes, seeds from a seminatural meadow, and application of fresh hay) and different frequencies of mowing (one, two, or three times per year). Soil invertebrates were identified to the taxa, using light microscopes. Richness and diversity indices were calculated, and a redundancy analysis was conducted. Generally, mowing intensity negatively influenced soil organisms, although increased mowing frequency positively affected the abundance of some taxa (Symphyla, Hemiptera). Mowing twice per year decreased the abundance of soil invertebrates, but not their diversity. Soil invertebrate taxa had the greatest abundance in the plots sown with a seed mixture containing grasses with legumes. Among the restoration methods studied, mowing once a year and introducing grasses with legumes represented the least harmful strategy with regard to soil invertebrate abundance. Further studies are needed to investigate the dynamics of soil mesofauna exposed to long-term mowing and changes in vegetation characteristics.

## Introduction

Plant invasions have serious negative effects on ecosystems and species diversity^1,2^ and can disrupt the linkages between the above- and belowground communities^3,4^. Goldenrods (*Solidago canadensis* L. and *Solidago gigantea* Aiton.) are among the most widespread invasive alien plants in Central Europe^5^. Invasive *Solidago* species can alter soil physicochemical properties (e.g., soil moisture, water holding capacity, organic carbon, total nitrogen content, available phosphorus, exchangeable cations) and cause biological changes in the soil (e.g., microbial biomass, respiration rate, nitrogen mineralization, soil enzyme activities)^2,6–8^. Moreover, *Solidago* invasion has had negative consequences in communities of springtails^7^, nematodes^9^, coleopterans^10,11^, ants^12–14^, and pollinators^15^.

The ecological restoration of lands invaded by alien plants may use herbicides, mowing, burning, and labour-intensive practices such as slashing or hand-felling and harrowing^16–18^. However, herbicides negatively affect nontarget species and the belowground community^18,19^. In addition, during restoration activities, seeding methods, seeding rates, and the use of a cover crop with native grasses respectively influence the disturbance, colonization, and nitrogen content of the soil^16,20^. Either individually or in combination, multiple forms of control strategies, such as manual removal, periodic flooding, grazing, scalping, mowing, rototilling, different seeding methods, turf stripping, and the use of herbicides have been investigated in recent studies on the ecological restoration of *Solidago*-invaded land^20–22^. Świerszcz et al*.* showed that herbicide (containing glyphosate) was beneficial for short-term eradication of invasive *Solidago* spp. and subsequent restoration of a meadow^21^. However, a 6-year experiment by Szymura et al*.* showed that herbicides were not effective for long-term removal of *Solidago* spp.^20^. In their study, adding fresh hay and mowing twice per year represented the best practice for restoring old fields invaded by *Solidago* spp.

Soil invertebrates inhabit the upper layer of soil and include medium-sized organisms (0.2–2 mm), such as most of the Collembola, Acari, Protura, and Nematoda, among others^23^. Soil invertebrates function as a community that supports major soil functions, such as the decomposition of organic matter and nutrient cycling^24^. Thus, changes in soil fauna, directly or indirectly, have impacts on soil functioning. Soil fauna communities and certain taxa or species serve as important indicators of soil health^25^.

Plant species composition can alter soil ecosystems by changing the structure of the habitat and its abiotic properties^2,7^. In addition, plant species composition can cause changes in soil invertebrate abundance and diversity. For example, both grasses and legumes have a beneficial effect on the density and diversity of Collembola^26^, while invasive plants reduce their density^7^.

Many restoration practices mainly focus on the aboveground components of ecosystems^27^. However, restoration ecology involves the integration of aboveground–belowground linkages, or plant–soil interactions, as well as the identification of effective intervention practices and the prediction of ecosystem recovery^3,27,28^. Although the impact of *Solidago* spp. on belowground soil components such as soil invertebrates has not been well documented^7^, it is known that soil microarthropods are sensitive to land management practices. For example, frequent mowing negatively affects soil organisms such as nematodes^29^, earthworms^30^, and bacteria^31^. Therefore, soil invertebrates can be used as bioindicators of biological soil quality through assessment of their rapid response to any changes in the soil environment^32–34^.

The current study aimed to evaluate how *Solidago* control methods and the use of different seed mixtures affected soil invertebrates in *Solidago*-invaded stands undergoing land restoration. We hypothesized that the abundance and composition of different soil invertebrates collected from these stands would vary according to the type of restoration method. From a practical point of view, we aimed to identify a method that effectively removes *Solidago*, while maintaining high biodiversity and abundance of soil invertebrates.

## Materials and methods

### Study site

The experiment was conducted on abandoned former agricultural land dominated by invasive North American *Solidago* spp. (*S. gigantea* and *S. canadensis*), at an altitude of 118 m a.s.l. The land is in a small river valley and is surrounded by suburban buildings and extensively used meadows in Wrocław, Poland (51°09′42.57″N, 17°06′43.97″E; elevation 116.4 m). The soil type is Anthropic Regosol, loamy sand texture. The mean annual temperature in the region is 9 °C, and the mean annual precipitation is 578.2 mm. Meteorological data for the period of 1968–2019 were obtained from the Agro- and Hydrometeorology Observatory in Swojczyce, Wrocław (51°06′56.6″N, 17°08′29.4″E).

### Field experimental design

**Figure 1:**
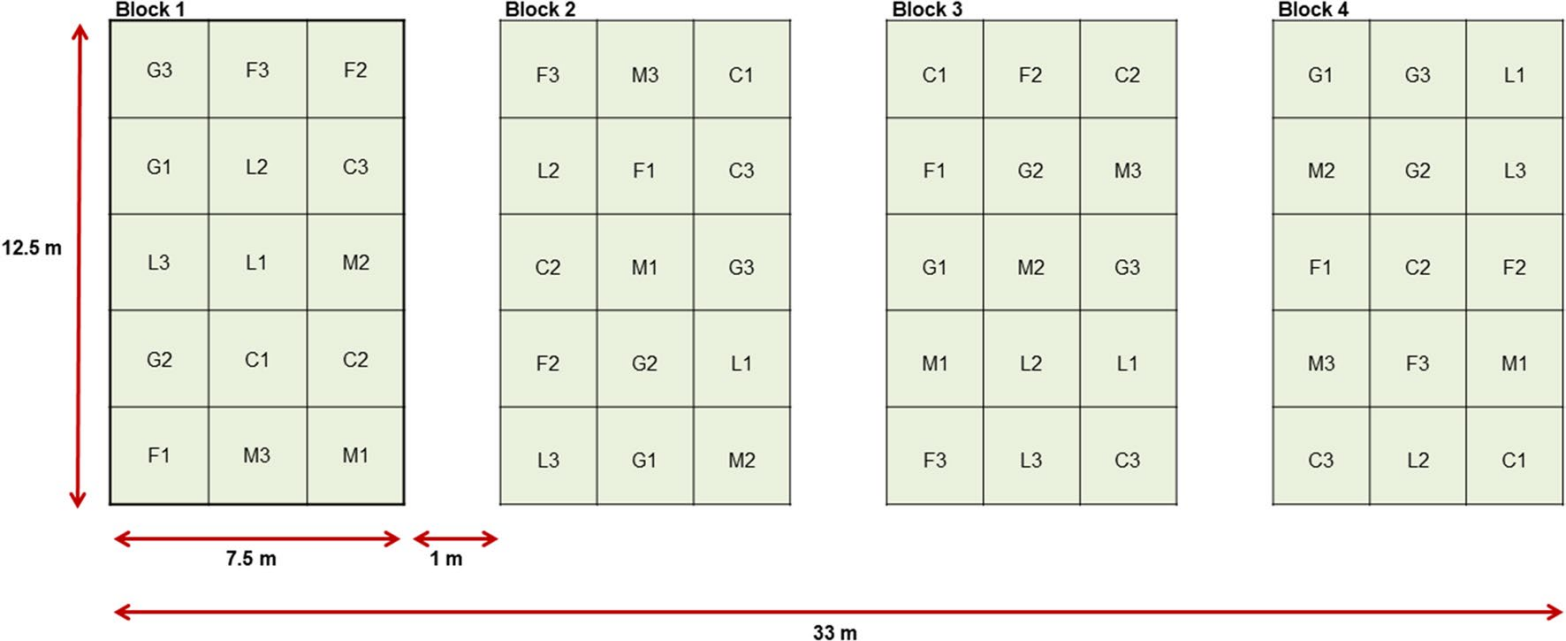
Experimental design based on sowing mixtures [grasses (G), grasses with legumes (L), seeds from the seminatural meadow (M), application of fresh hay (H), and control (C)] and mowing regimes [once (1), twice (2), and three times (3)].

The field experiment on *Solidago* species removal and land reclamation was established in April 2020. The experiment used a 5 × 3 factorial arrangement in a completely randomized design with four replications, as shown in Fig. 1. The four blocks, each containing 15 plots (2.5 × 2.5 m), were established with a separation of 1 m from each other. The entire experimental area was mowed, and the soil was then prepared for seeding, using a rototiller followed by a power harrow. After the area was seeded, it was compacted with a roller. Two experimental factors were used: (1) various methods of seed introduction and seed composition (sowing mixtures: grasses, grasses with legumes, seeds collected from the seminatural meadow, application of fresh hay and without seed application) (Table 1), and (2) different frequencies of mowing (one, two, or three times per year). The species composition of seed mixtures and fresh hay are presented in Supplementary Table S1. In both 2020 and 2021, the plots were mown according to the planned scheme: once (June), twice (June and August), or three times (June, August, and September).

**Table 1:** Various methods of seed introduction and composition (The species composition of seed mixtures and fresh hay are presented in Supplementary Table S1).

Sowed mixtures	Description
	Conventional mixture of four grass species used for grassland establishment
	Four grass species (the same used in the G mixture), along with two clover species
	A mixture of 37 plant species typically occurring in seminatural grasslands. Rieger-Hofmann GmbH distributes the specialized seed mixture, which is produced using seed mixtures of native species with a controlled geographical origin
	Fresh hay was collected at the Experimental Station of Wrocław University of Environmental and Life Sciences in Radomierz (50°54ʹ15.1″N, 15°53ʹ58.8″E), Silesia, Poland, Central Europe. The station is located in the sub-mountain area, Kaczawskie Mountains. The pastures are grazed for the entire growing season, and rotation pasturage is used, with sporadic mowing. During the field observation in June 2020, 47 species were observed. The fresh hay was collected and spread in July 2020
	No seed application

### Material collection and preparation

A single sample of soil was collected from the centre of each plot with the use of a 10-cm-diameter circular sampler, at a depth of 10 cm in autumn (September 2020 and August 2021), spring (April 2021), and summer (June 2021), respectively. The samples were collected within 2 × 2 m areas inside each experimental plot. The soil samples were illuminated in Tullgren funnels (25 W light bulb) for 24 h. Soil organisms extracted from the soil were kept in 75% alcohol until their identification according to taxa. The soil organisms were identified according to the Soil Invertebrate Key of the Ecological Society of America^35^.

### Data analysis

The richness and diversity indices of the total fauna communities were calculated. The Shannon–Weaver index (Hʹ) was calculated according to the following formula^36^:

$$H^{\prime } = - \mathop \sum \limits_{i = 1}^{R} p_{i} \ln p_{i}$$

where *p*_*i*_ is the proportion of individuals belonging to the *i*th taxa and R is the total number of species. The Pielou index (J) was calculated according to the formula^37^:

$$J = \frac{{H^{\prime } }}{\ln \left( S \right)}$$

where Hʹ is the Shannon–Weaver index and *S* is the total number of species in a sample.

The Margalef species richness index (D) was calculated according to the following formula^38^:

$$D = \frac{S - 1}{{\ln \left( N \right)}}$$

where *S* is the total number of species in a sample and *N* is the total number of individuals in the sample.

Statistical analysis was done with SAS University Edition (version 9.0), using the generalized linear mixed model (GLMM) with repeated measurements. The explanatory variables were mowing, seed introduction method, season, and their interactions. The repeated factor was the season, and the random factor was the block. Significant differences between treatments were revealed using the Tukey HSD test (*p* ≤ 0.05). The invertebrate taxa were correlated with the environmental factors by using redundancy analysis (RDA) performed in Canoco 5.0. The data were log-transformed before analysis. Only taxa that occurred in at least three samples were included in the analysis.

### Ethics approval

The experimental research and field studies on plants, including the collection of plant material, complied with the relevant institutional, national, and international guidelines and legislation.

## Results

### Community responses

**Figure 2:**
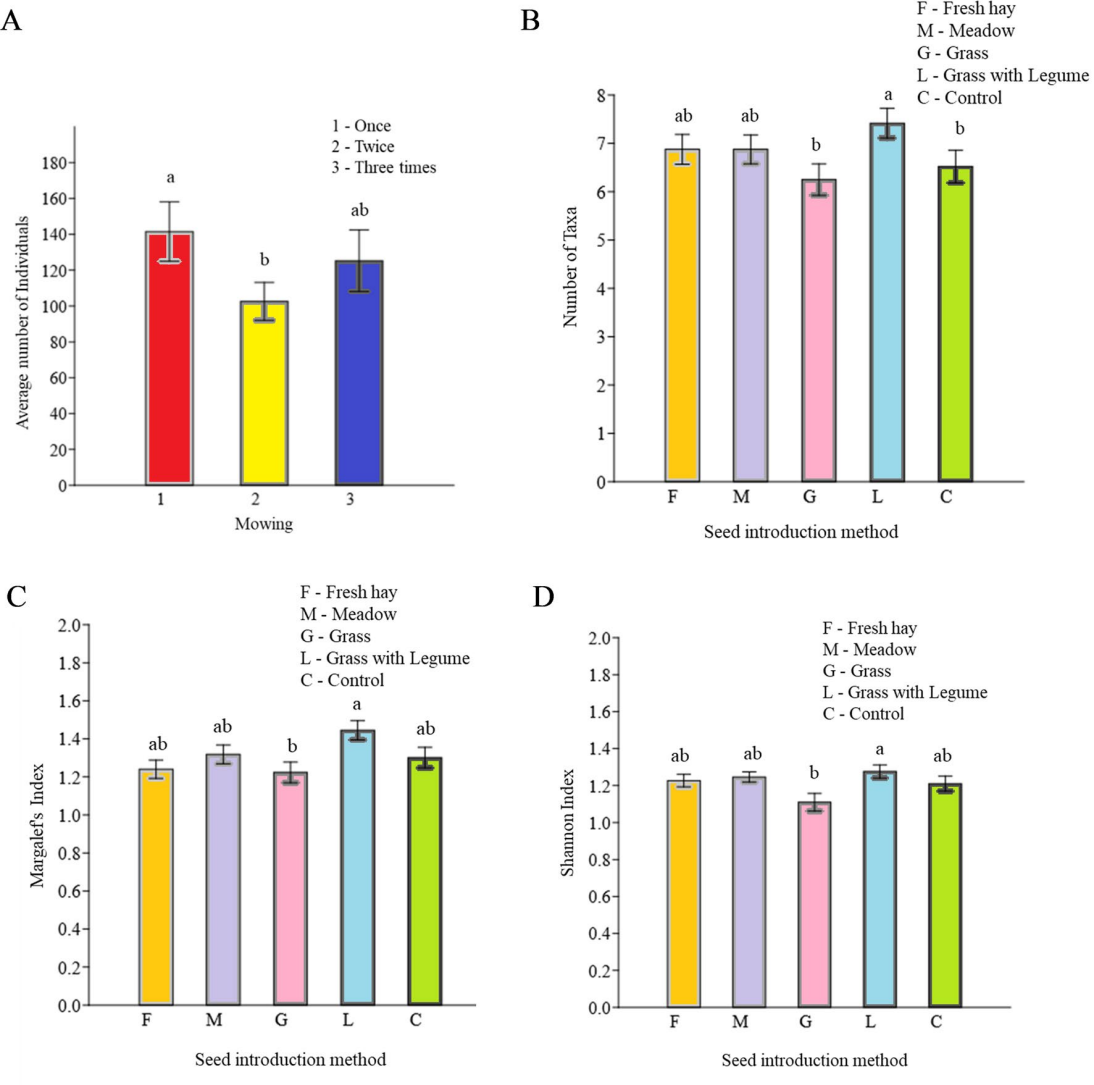
The effects of mowing and seed introduction methods on the community indices of soil invertebrates and their demographic responses. The different lowercase letters indicate significant differences between treatments, *p *≤ 0.05.

Both experimental factors, the seed composition and introduction method and the mowing regimes, significantly affected the soil invertebrates (Table 2). The average number of all organisms was significantly higher in plots mowed once compared with plots mowed twice (Fig. 2A), and the diversity indices differed significantly between seed introduction methods (Table 2). The number of taxa was higher in plots where the mixture of grass with legumes was sown (L), in comparison with control (C) and grass mixture (G) plots (Fig. 2B). In addition, two diversity indices (Shannon Weaver and Margalef) showed that higher diversity was associated with legumes (L) relative to other treatments, particularly the use of grass species (G) (Fig. 2C,D). The interactions of seed × mowing, seed × season, and mowing × season did not yield significant differences, while season significantly affected abundance, number of taxa, and both diversity indices (Table 2).

**Table 2:** The statistical summary of the effects of experimental treatments on the community indices of soil invertebrates.

	Seed		Mowing		Season		Seed × Mowing		Seed × Season		Mowing × Season	
df	4		2		3		8		12		6	
	F	p	F	p	F	p	F	p	F	p	F	p
Abundance	0.35	0.84	3.84	**0.02**	29.28	** < 0.001**	1.70	0.10	0.77	0.68	0.58	0.75
Number of taxa	3.45	**0.01**	2.95	0.06	57.43	** < 0.001**	0.94	0.49	1.04	0.42	1.99	0.07
Shannon–Weaver	2.73	**0.03**	0.13	0.87	9.24	** < 0.001**	0.82	0.59	1.35	0.19	0.41	0.87
Pielou	0.68	0.60	1.03	0.35	57.00	** < 0.001**	0.65	0.74	1.33	0.20	0.36	0.90
Margalef	2.88	**0.02**	0.72	0.49	8.08	** < 0.001**	0.86	0.55	0.32	0.34	1.75	0.11

Bold values indicate significant effects.

### Taxa responses

**Figure 3:**
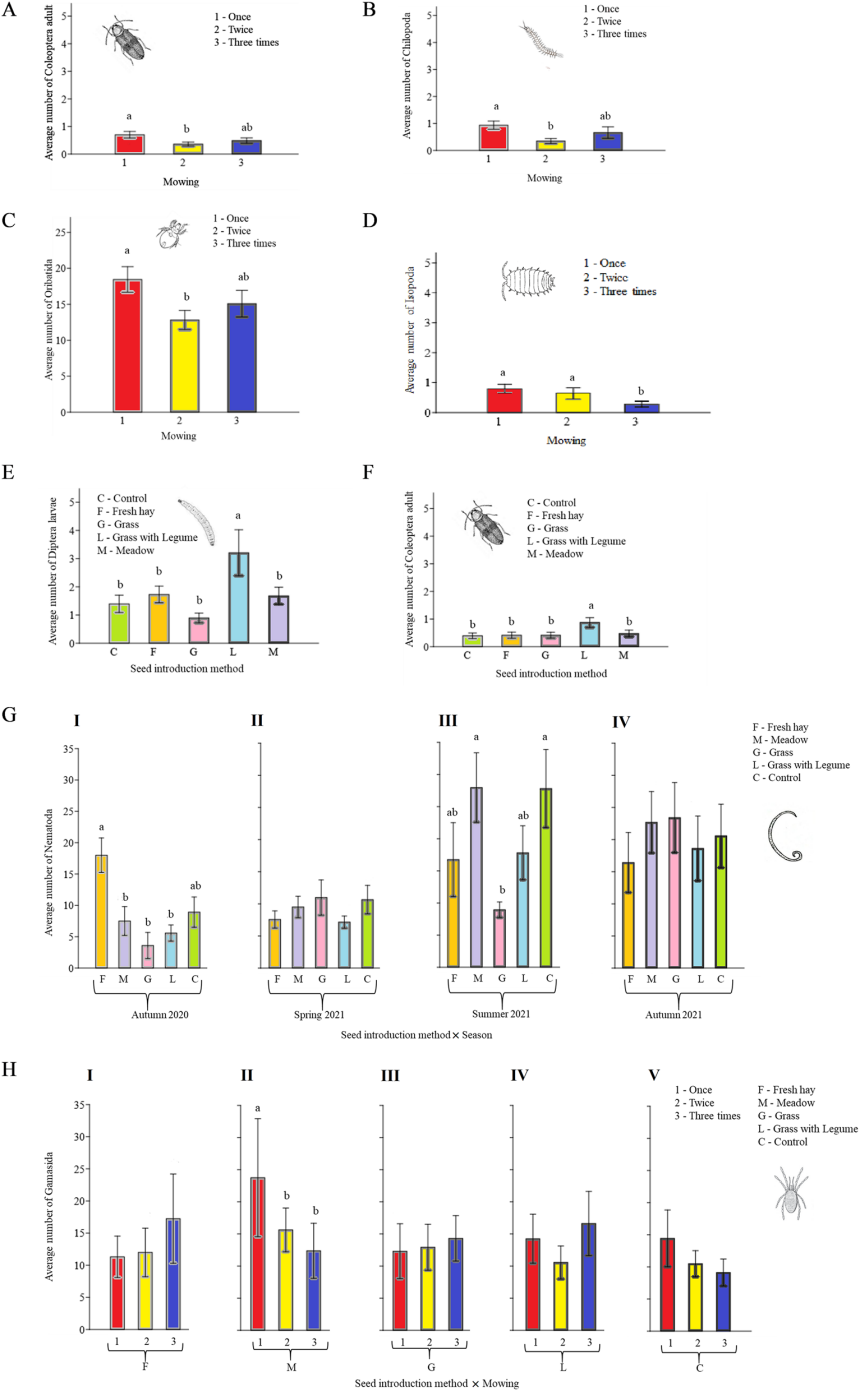
The effects of mowing and seed introduction methods, as well as their interactions, on the abundance of taxa. The different lowercase letters indicate significant differences between treatments; mowing regimes (**A**–**D**) and seed introduction method (**E**,**F**), *p* ≤ 0.05. The significance of differences is shown within the particular groups: (**G**) season and (**H**) seed introduction method, *p* ≤ 0.05.

Among all factors, mowing had the greatest impact on the abundance of all soil invertebrate taxa (Table 3). Mowing once positively affected Isopoda, Chilopoda, Oribatida, Gamasida, and adult Coleoptera abundance (Fig. 3A,B,C,D,H). Seed introduction methods significantly affected Diptera larvae and Coleoptera adults (Table 3). In both cases, the mean number of organisms was higher in plots where grasses with legumes (L) were sown compared with other seed mixtures (Fig. 3E,F), and there were no differences between other seed composition and introduction methods. The differences in Nematoda abundance were significant for seed composition and introduction method, as well as season (Fig. 3G). Season was significant for all taxa except Diptera larvae (Table 3).

**Table 3:** The statistical summary of the effects of experimental treatments and their interactions on abundance of the soil invertebrates.

Soil Invertebrates	Seed		Mowing		Season		Seed × Mowing		Seed × Season		Mowing × Season	
df	4		2		3		8		12		6	
	F	p	F	p	F	p	F	p	F	p	F	p
Astigmata	1.15	0.33	0.24	0.79	6.22	**< 0.001**	0.84	0.57	1.33	0.20	0.70	0.65
Chilopoda	0.77	0.54	4.07	**0.02**	15.04	**< 0.001**	1.90	0.06	0.80	0.65	1.24	0.29
Collembola	0.60	0.66	2.12	0.12	31.02	**< 0.001**	1.66	0.11	1.03	0.43	0.68	0.66
Gamasida	1.06	0.38	0.81	0.45	45.31	**< 0.001**	2.58	**0.01**	0.71	0.74	1.05	0.39
Isopoda	1.23	0.30	3.36	**0.04**	10.32	**< 0.001**	0.77	0.63	1.01	0.44	2.05	0.06
Oribatida	0.79	0.53	3.47	**0.03**	18.84	**< 0.001**	0.35	0.94	0.63	0.82	1.95	0.07
Nematoda	1.71	0.15	0.17	0.85	14.11	**< 0.001**	1.10	0.37	2.60	**0.00**	0.67	0.68
Symphyla	1.63	0.17	0.05	0.95	15.18	**< 0.001**	1.56	0.14	0.65	0.80	1.73	0.12
Arachnida	0.40	0.81	0.33	0.72	8.74	**< 0.001**	0.95	0.48	0.54	0.89	0.80	0.57
Diptera larvae	4.02	**0.00**	0.16	0.85	4.33	0.055	0.57	0.80	1.05	0.40	1.68	0.13
Coleoptera adult	2.91	**0.02**	3.44	**0.03**	11.76	**< 0.001**	0.37	0.94	0.72	0.73	0.90	0.49
Coleoptera larvae	1.27	0.28	0.43	0.65	8.82	**< 0.001**	0.71	0.68	0.43	0.95	0.25	0.96

Bold values indicate significant effects.

**Figure 4:**
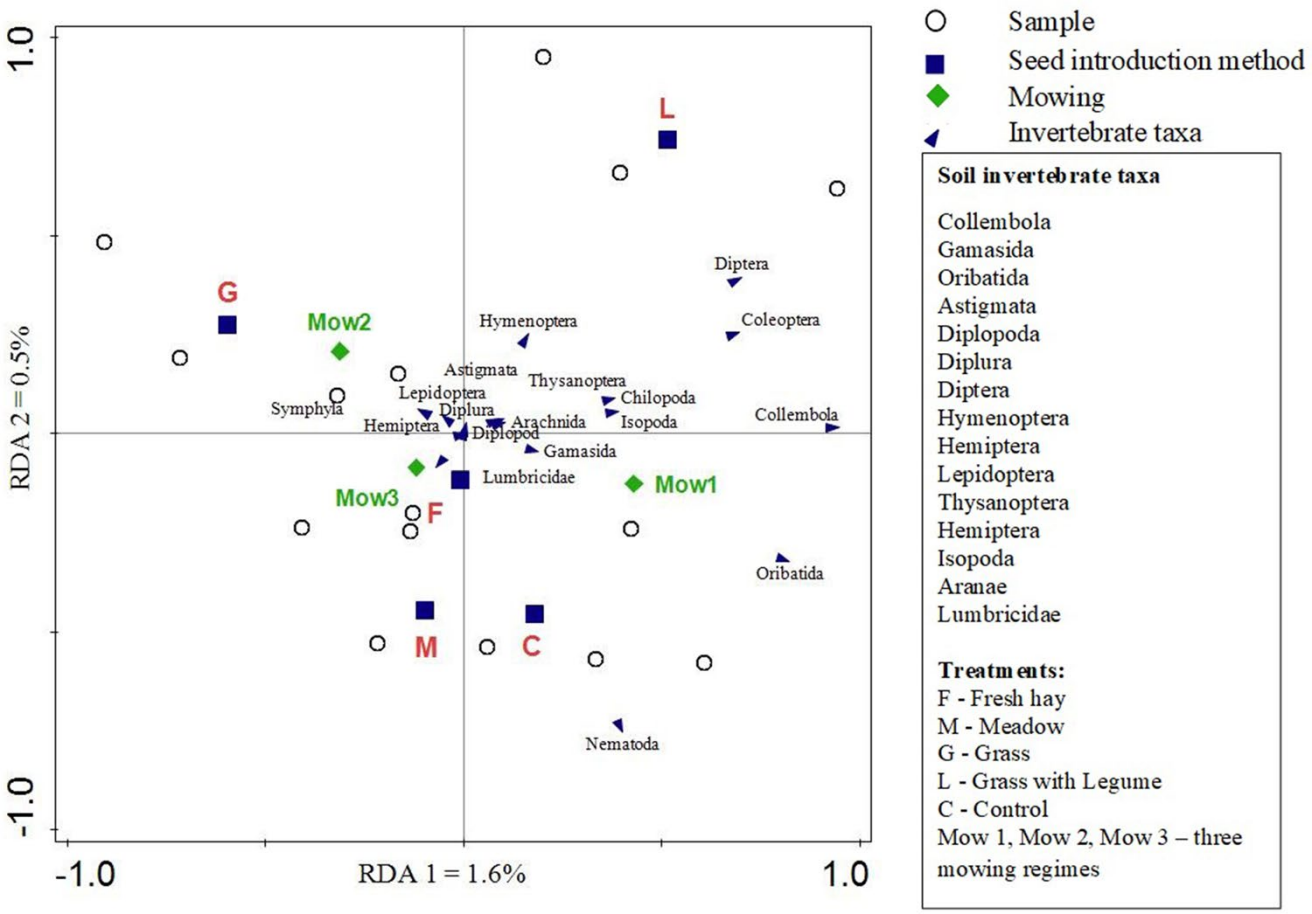
The redundancy analysis biplot of soil invertebrate community in association with mowing, seed introduction methods, and season.

Redundancy analysis (Fig. 4) was used to compare the relative effects of mowing and the seed composition and introduction method on different taxa simultaneously (see the results of redundancy analysis in Supplementary Table S2). The abundance of most of the soil invertebrate taxa had positive correlations with the grass with legumes mixture and mowing once per season. The abundance of a few taxa (Symphyla, Hemiptera) was positively associated with greater mowing frequency. The effects of the seed introduction method were less distinct. Most of the taxa (e.g., Isopoda, Collembola, Chilopoda) were positively oriented to the grass with legumes mixture (L), particularly in comparison with grass and the control.

## Discussion

Land restoration methods are often applied to large areas, and they should not only be effective but also safe for the environment^39^. It is important to assess the risk related to the application of land restoration methods with regard to both the aboveground impacts and the belowground effects. A useful tool for assessing changes in the soil environment is the analysis of soil fauna occurrence and diversity. The indicators based on soil fauna are related to soil functionality and further plant growth^40^. Soil fauna mainly supports the decomposition of soil organic matter and affects the turnover of nutrients^41^. The presence of these organisms is closely related to the soil structure and the chemical properties of the soil, and any changes in soil conditions could have an impact on this group of organisms^42^.

We found that high mowing frequency negatively affected soil fauna abundance. This study tested three mowing regimes (one, two, and three times a year) and revealed that mowing once per year positively affected soil fauna. This finding is in line with the study of Zhao et al., in which nematode abundance decreased under more frequent mowing, from once per year (high intensity) to once every 6–12 years (low frequency)^29^. In addition, unmown meadows were more beneficial for earthworms relative to meadows mown once a year^30^.

Van Eekeren et al. concluded that mowing without tillage is one of the best practices for restoring the soil micro-arthropods and ecosystem services in permanent grasslands^43^. Mowing techniques (e.g., the type of mowing heads and mower) can also have a considerable impact arthropod fauna^44,45^. However, Hyvönen et al. found that mown meadows had higher abundances of pollinators such as bumblebees and honeybees^30^. The effect of mowing on the belowground ecosystem is generally unknown and needs further study. We suggest that mowing indirectly affects soil fauna through changes in soil conditions. Soil fauna is closely related to soil functions and microbial activity^46^, and mowing negatively affects soil conditions, such as temperature, moisture, and carbon sequestration^47^, as well as microbial activity^31^. In a study in Mongolia, the mowing of grasslands had an extreme effect on the upper soil layer, as shown by a 4 °C increase in the soil temperature and a 47% decrease in the soil moisture^47^. Such negative effects on soil conditions can further affect soil fauna. Although mowing is beneficial for maintaining grassland vegetation, we recommend limiting this treatment to avoid disrupting the belowground ecosystem.

In our study, we removed the aboveground biomass during mowing, which may have reduced soil invertebrate abundance. Grass harvesting is the traditional way of grasslands management all over the world, which consequently causes biomass removal and reduction of the resources for decomposers^31,47^. However, leaving the cuttings did not mitigate the negative effect of mowing on earthworms in comparison with non-mown areas^30^. In addition, the thickness and origin of the litter can have an impact on the abundance of soil invertebrates such as springtails, nematodes, and mites^48,49^. For instance, knotweed litter negatively affected Collembola, because of its extended decomposition time and slower nutrient release compared with native species^48^.

The current study also described a direct influence between seasons and soil invertebrates, and seasonal reliance on plant community characteristics was associated with the soil invertebrate community. In particular, Collembola abundance changed during the season, which may have been related to the sensitivity of those organisms to soil moisture and temperature^50^. The seasonal changes in earthworm communities in grassland ecosystem were previously explained by the availability and quality of organic resources during each season, which is the probable explanation for similar observations in our study^51^.

According to Eisenhauer et al. and Hyvönen et al., the presence of various plant functional groups differentially affects the densities of particular soil invertebrates and their consistency over time^30,51,52^. Moreover, ample evidence indicates that legumes have positive effects on ecosystem functioning^53,54^. The cultivation of legumes improves the soil nitrogen level and primary productivity, as well as carbon sequestration^53^. In addition, legumes increase the bacterial activity in the soil^55^ and enhance the complexity of the trophic links in the soil food web^56^. Legumes’ ability to create specific root systems can also change the diversity of soil fauna^57^. Furthermore, the introduction of legumes can increase ecosystem resistance to plant invasion^58^. In the present study, the introduction of legumes with grass seeds positively affected soil invertebrates’ abundance and diversity, which accorded with the findings of previous research^59–62^. The results also showed that the introduction of grass only or no introduction of seeds (control) was not beneficial for soil fauna.

Study findings indicated that both mowing and aboveground plant diversity significantly affected soil fauna. These effects have several possible explanations, and further research is needed to test them. The most probable explanation is that changes in soil conditions had an indirect effect on soil fauna. Nevertheless, the soil fauna is an effective indicator of grassland management and restoration success.

## Conclusions

Our results demonstrate that a greater intensity of mowing has a negative impact on soil organisms. Mowing twice a season decreased the abundance of invertebrate taxa. The application of a seed mixture of grass with legumes increased the diversity of soil invertebrates compared with other seed composition and introduction methods. The mixture of legumes and grass seeds was also beneficial for the abundance of most invertebrate taxa. Mowing once a season and the introduction of a mixture of grasses with legumes constitute the most suitable method for restoring *Solidago*-invaded stands, while also maintaining soil invertebrate abundance.

## Supplementary Information

Supplementary Information.

## Data Availability

The data that support the findings of this study are available from the corresponding author upon request.
