# Supplementary material for: The impact of restoration methods for Solidago-invaded land on soil invertebrates
Source: Sci Rep. 2022 Oct 5;12:16634. doi: 10.1038/s41598-022-20812-5 (PMC9534866; doi:10.1038/s41598-022-20812-5)
Supplement: Supplementary file 1 — Supplementary Information. [file 41598_2022_20812_MOESM1_ESM.docx]

**Table S1.** Species composition of seed introduction methods

| Meadow seed mixture (M) | Seed % | Grass seeds (G) | Seed % | Grasses with legumes (L) | Seed % | Fresh hay (F) | Coverage % |
| --- | --- | --- | --- | --- | --- | --- | --- |
| *Achillea millefolium* L. | 1 .2 | *Festuca pratensis* Huds. | 50.0 | *Festuca pratensis* Huds. | 30.0 | *Achillea millefolium* L. | 7.0 |
| *Agrostis capillaris* L. | 2 .0 | *Lolium perenne* L. | 30.0 | *Lolium perenne* L. | 20.0 | *Aegopodium podagraria* L. | 0.1 |
| *Alopecurus pratensis* L. | 2 .0 | *Phleum pratense* L*.* | 10.0 | *Phleum pratense* L. | 10.0 | *Agrostis capillaris* L. | 12.0 |
| *Anthoxanthum odoratum* L. | 3 .0 | *Poa pratensis* L. | 10.0 | *Poa pratensis* L. | 10.0 | *Alchemilla monticola* Opiz | 2.0 |
| *Anthriscus sylvestris* (L.) Hoffm. | 1 .5 |  |  | *Trifolium pratense* L. | 10.0 | *Alopecurus pratensis* L*.* | 0.5 |
| *Arrhenatherum elatius* (L.) J.Presl & C.Presl | 2 .0 |  |  | *Trifolium repens* L. | 20.0 | *Anthoxanthum odoratum* L. | 5.0 |
| *Avenula pubescens* (Huds.) Dumort. | 2 .0 |  |  |  |  | *Anthriscus sylvestris* (L.) Hoffm. | 0.5 |
| *Bromus hordeaceus* L. | 3 .0 |  |  |  |  | *Arrhenatherum elatius* (L.) J.Presl & C.Presl | 7.0 |
| *Centaurea jacea* L. | 1 .5 |  |  |  |  | *Campanula patula* L. | 0.5 |
| *Crepis biennis* L. | 1 .0 |  |  |  |  | *Centaurea jacea* L. | 3.0 |
| *Cyanus segetum* Hill | 2 .6 |  |  |  |  | *Cerastium fontanum* Baumg. | 0.5 |
| *Cynosurus cristatus* L. | 4 .0 |  |  |  |  | *Chaerophyllum aromaticum* L. | 8.0 |
| *Dactylis glomerata* L. | 2 .0 |  |  |  |  | *Dactylis glomerata* L. | 5.0 |
| *Daucus carota* L. | 2 .0 |  |  |  |  | *Dianthus deltoides* L. | 1.0 |
| *Festuca pratensis* Huds. | 7 .0 |  |  |  |  | *Elymus repens* (L.) Gould | 0.1 |
| *Festuca rubra* L. | 22 .0 |  |  |  |  | *Festuca pratensis* Huds. | 2.0 |
| *Galium verum* subsp. wirtgenii (F. W. Schultz) Oborný | 0 .5 |  |  |  |  | *Festuca rubra* L. | 1.0 |
| *Galium album* Mill. | 2 .0 |  |  |  |  | *Galium mollugo* L. | 3.0 |
| *Heracleum sphondylium* L. | 0 .5 |  |  |  |  | *Geum urbanum* L. | 0.5 |
| *Knautia arvensis* (L.) DC. | 0 .8 |  |  |  |  | *Heracleum sphondylium* L. | 1.0 |
| *Leontodon hispidus* L. | 0 .3 |  |  |  |  | *Holcus lanatus* L. | 5.0 |
| *Leucanthemum vulgare* Lam. | 3 .0 |  |  |  |  | *Hypericum maculatum* Crantz | 1.5 |
| *Lolium perenne* L. | 5 .0 |  |  |  |  | *Jacobaea vulgaris* Gaertn. | 0.1 |
| *Lotus corniculatus* L. | 1 .0 |  |  |  |  | *Knautia arvensis* (L.) DC. | 1.0 |
| *Lychnis flos-cuculi* L. | 0 .5 |  |  |  |  | *Lathyrus pratensis* L. | 2.0 |
| *Papaver rhoeas* L. | 1 .5 |  |  |  |  | *Leucanthemum vulgare* Lam. | 2.0 |
| *Plantago lanceolata* L. | 2 .8 |  |  |  |  | *Lolium perenne* L. | 0.1 |
| *Poa angustifolia* L. | 13 .0 |  |  |  |  | *Lotus corniculatus* L. | 0.5 |
| *Prunella vulgaris* L. | 1 .5 |  |  |  |  | *Lychnis flos-cuculi* L. | 0.5 |
| *Rumex acetosa* L. | 0 .5 |  |  |  |  | *Phleum pratense* L. | 5.0 |
| *Sanguisorba officinalis* L. | 0 .4 |  |  |  |  | *Pimpinella saxifraga*L. | 0.5 |
| *Scorzoneroides autumnalis* (L.) Moench | 0 .2 |  |  |  |  | *Plantago lanceolata* L. | 1.5 |
| *Silene dioica* (L.) Clairv. | 1 .8 |  |  |  |  | *Poa pratensis* L. | 6.0 |
| *Silene vulgaris* (Moench) Garcke | 1 .5 |  |  |  |  | *Ranunculus acris* L. | 0.5 |
| *Tragopogon pratensis* L. | 0 .8 |  |  |  |  | *Ranunculus repens* L. | 1.0 |
| *Trifolium pratense* L. | 0 .6 |  |  |  |  | *Rumex acetosa* L. | 1.0 |
| *Trisetum flavescens* (L.) P.Beauv. | 3 .0 |  |  |  |  | *Solidago virgaurea* L. | 0.1 |
|  |  |  |  |  |  | *Stellaria graminea* L. | 2.0 |
|  |  |  |  |  |  | *Taraxacum officinale* (L.) F.H.Wigg | 0.1 |
|  |  |  |  |  |  | *Tragopogon pratensis* L. | 0.1 |
|  |  |  |  |  |  | *Trifolium pratense* L. | 0.5 |
|  |  |  |  |  |  | *Trifolium repens* L. | 2.0 |
|  |  |  |  |  |  | *Trisetum flavescens* (L.) P.Beauv. | 5.0 |
|  |  |  |  |  |  | *Veronica chamaedrys* L. | 7.0 |
|  |  |  |  |  |  | *Vicia cracca* L. | 0.5 |
|  |  |  |  |  |  | *Vicia hirsuta* (L.) Gray | 1.0 |
|  |  |  |  |  |  | *Vicia sepium* L. | 1.0 |

**Table S2.** The results of RDA analysis on taxa of soil invertebrates in relation to environmental treatments.

|  | Axis 1 | Axis 2 | Axis 3 | Axis 4 |
| --- | --- | --- | --- | --- |
| Eigenvalues | 0.0160 | 0.0050 | 0.0042 | 0.0022 |
| Explained variation (cumulative) | 1.60 | 2.10 | 2.52 | 2.74 |
| Pseudo-canonical correlation | 0.2171 | 0.2220 | 0.3098 | 0.1678 |
| Explained fitted variation (cumulative) | 53.44 | 70.28 | 84.39 | 91.88 |
